# Clinician perceptions of a clinical decision support system to reduce cardiovascular risk among prediabetes patients in a predominantly rural healthcare system

**DOI:** 10.1186/s12911-022-02032-z

**Published:** 2022-11-19

**Authors:** Daniel M. Saman, Clayton I. Allen, Laura A. Freitag, Melissa L. Harry, JoAnn M. Sperl-Hillen, Jeanette Y. Ziegenfuss, Jacob L. Haapala, A. Lauren Crain, Jay R. Desai, Kris A. Ohnsorg, Patrick J. O’Connor

**Affiliations:** 1 Carle Foundation Hospital Clinical Business and Intelligence, 611 W Park Street, Urbana, IL 61801 USA; 2 Essentia Institute of Rural Health, 502 E. Second Street, 6AV-2, Duluth, MN 55805 USA; 3 HealthPartners Institute, 3311 E. Old Shakopee Road, Bloomington, MN 55425 USA; 4 Minnesota Department of Health, 85 East 7Th Place, St. Paul, MN 55164 USA

**Keywords:** Clinical decision support, Electronic medical record, Primary care clinician, Advanced practice provider, Cardiovascular disease, Cardiovascular risk, Hypertension, Diabetes, Dyslipidemia, Prediabetes

## Abstract

**Background:**

The early detection and management of uncontrolled cardiovascular risk factors among prediabetes patients can prevent cardiovascular disease (CVD). Prediabetes increases the risk of CVD, which is a leading cause of death in the United States. CVD clinical decision support (CDS) in primary care settings has the potential to reduce cardiovascular risk in patients with prediabetes while potentially saving clinicians time. The objective of this study is to understand primary care clinician (PCC) perceptions of a CDS system designed to reduce CVD risk in adults with prediabetes.

**Methods:**

We administered pre-CDS implementation (6/30/2016 to 8/25/2016) (n = 183, 61% response rate) and post-CDS implementation (6/12/2019 to 8/7/2019) (n = 131, 44.5% response rate) independent cross-sectional electronic surveys to PCCs at 36 randomized primary care clinics participating in a federally funded study of a CVD risk reduction CDS tool. Surveys assessed PCC demographics, experiences in delivering prediabetes care, perceptions of CDS impact on shared decision making, perception of CDS impact on control of major CVD risk factors, and overall perceptions of the CDS tool when managing cardiovascular risk.

**Results:**

We found few significant differences when comparing pre- and post-implementation responses across CDS intervention and usual care (UC) clinics. A majority of PCCs felt well-prepared to discuss CVD risk factor control with patients both pre- and post-implementation. About 73% of PCCs at CDS intervention clinics agreed that the CDS helped improve risk control, 68% reported the CDS added value to patient clinic visits, and 72% reported they would recommend use of this CDS system to colleagues. However, most PCCs disagreed that the CDS saves time talking about preventing diabetes or CVD, and most PCCs also did not find the clinical domains useful, nor did PCCs believe that the clinical domains were useful in getting patients to take action. Finally, only about 38% reported they were satisfied with the CDS.

**Conclusions:**

These results improve our understanding of CDS user experience and can be used to guide iterative improvement of the CDS. While most PCCs agreed the CDS improves CVD and diabetes risk factor control, they were generally not satisfied with the CDS. Moreover, only 40–50% agreed that specific suggestions on clinical domains helped patients to take action. In spite of this, an overwhelming majority reported they would recommend the CDS to colleagues, pointing for the need to improve upon the current CDS.

*Trial registration*: NCT02759055 03/05/2016.

## Background

While prediabetes is sometimes viewed as a minor health condition, it increases the risk of developing type 2 diabetes and many other conditions associated with diabetes, such as stroke, multiple diseases involving the eyes, nervous system, or kidneys, or being diagnosed with cardiovascular (CV) or coronary heart disease [1–3]. While 1 in 3 Americans meet conditions for prediabetes [1, 3], just 16% of those are aware they have prediabetes [1].

Periodic testing for abnormal glucose levels [2] in adults is recommended based on age, Body Mass Index (BMI), and other risk factors. Primary Care Clinicians (PCCs) play an important role in identifying patients with prediabetes, assessing CV risk, and delaying or preventing diabetes onset; however, glucose screening and follow-up of elevated fasting glucose values is often incomplete due to the nature of compressed patient visits, competing demands, fragmented care, inefficient health information systems, lack of accountability, and lack of point-of-care clinical decision support (CDS) [4–7].

To exacerbate things, focusing on diabetes prevention is often not a high clinical priority in busy primary care practices, and PCCs often have limited interest or skill in behavior change science or patient education, which are prominent components of prediabetes care in most patients [8]. Algorithm-based CDS tools may offer great assistance to PCCs by enhancing identification and management of prediabetes and guiding CV risk factor control in such patients, as has been the case with prostate and other cancers, particularly when PCCs need to discuss screening options [9–11]. Current evidence is lacking regarding PCCs’ usage or lack of usage of algorithm-based decision tools as well as their perceptions on the utility of CDS, and how such tools may support CV risk reduction among prediabetes patients.

Algorithmically-driven decision support tools have been found useful in many non-medical disciplines and often outperform expert judgment [12, 13], yet optimal utilization of these tools in primary care and other health care settings remains aspirational. For example, Saleem et al. [14] found six common barriers to CDS integration including “receiving and documenting ‘outside’ exam results, inaccuracy of the CDS, compliance issues, poor usability, lack of coordination between primary care and gastroenterology, and the need to attend to more urgent patient issues”. An earlier study (1998) concluded that CDS systems were highly promising and that the quality of studies were improving; however, they also reported that the effects of patient outcomes had not been sufficiently studied [15]. Very early computer assisted support in the emergency room suggested some promise for optimizing drug administration [16]. However, few studies to date have investigated PCC attitudes towards CDS systems for cardiometabolic care, and CDS use and effectiveness in outpatient chronic disease care remains inconsistent [17, 18].

### Objective and hypothesis

This cross-sectional study of PCCs aims to: (1) improve our understanding of PCCs' experience in delivering care to adult patients with prediabetes and one or more uncontrolled CVD risk factors, and (2) assess intervention clinic PCCs' overall satisfaction with the CDS system and satisfaction with specific aspects of the CDS system, and (3) assess intervention clinic PCCs' perceptions of CDS impact on shared decision making with patients.

## Methods

### Study participants

There were a total of 299 (pre-implementation) and 294 (post-implementation) Essentia Health PCCs from 36 primary care clinics included in a randomized control trial of an electronic medical record (EMR)-linked web-based CDS tool referred to as the *Wizard*. The CDS tool was designed to improve cardiovascular care for adults with prediabetes and one or more uncontrolled CVD risk factors. For the present study, the Essentia Health PCCs were invited to complete two cross-sectional electronic surveys. Essentia Health’s integrated healthcare system serves a wide and rural population in Minnesota, North Dakota, and Wisconsin with 14 hospitals and 71 clinics. The clinic-cluster randomized trial included PCCs who were either physicians (family practice or internal medicine) or advanced care practitioners (adult, pediatric, family, or geriatric nurse practitioners or physician assistants) practicing in one of the 24 intervention or 12 control primary care clinics. No compensation was provided for survey completion. PCCs who did not respond or who reported seeing patients in these clinics less than 3 days per week or were missing data on this question were excluded.

### Intervention

**Fig. 1:**
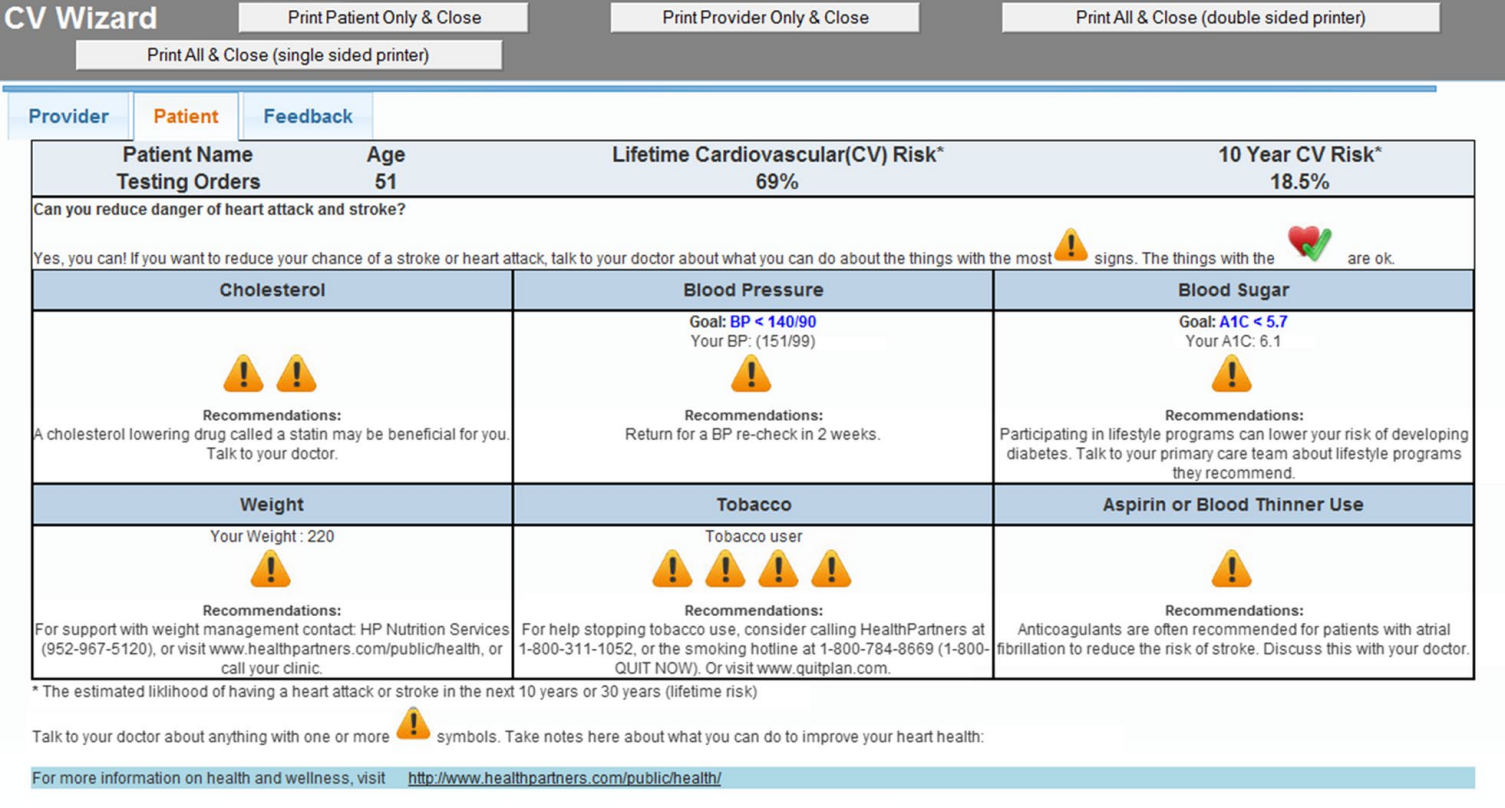
Patient view and printout of the clinical decision support system. Reprinted from Contemporary Clinical Trials, 114, Desai J, Saman D, Sperl-Hillen JM, Pratt R, Dehmer SP, Allen C, Ohnsorg K, Wuorio A, Appana D, Hitz P, Land A, Sharma R, Wilkinson L, Crain AL, Crabtree BF, Bianco J, O'Connor PJ. Implementing a prediabetes clinical decision support system in a large primary care system: Design, methods, and pre-implementation results, 106,686, Copyright (2022), with permission from Elsevier [20]

**Fig. 2:**
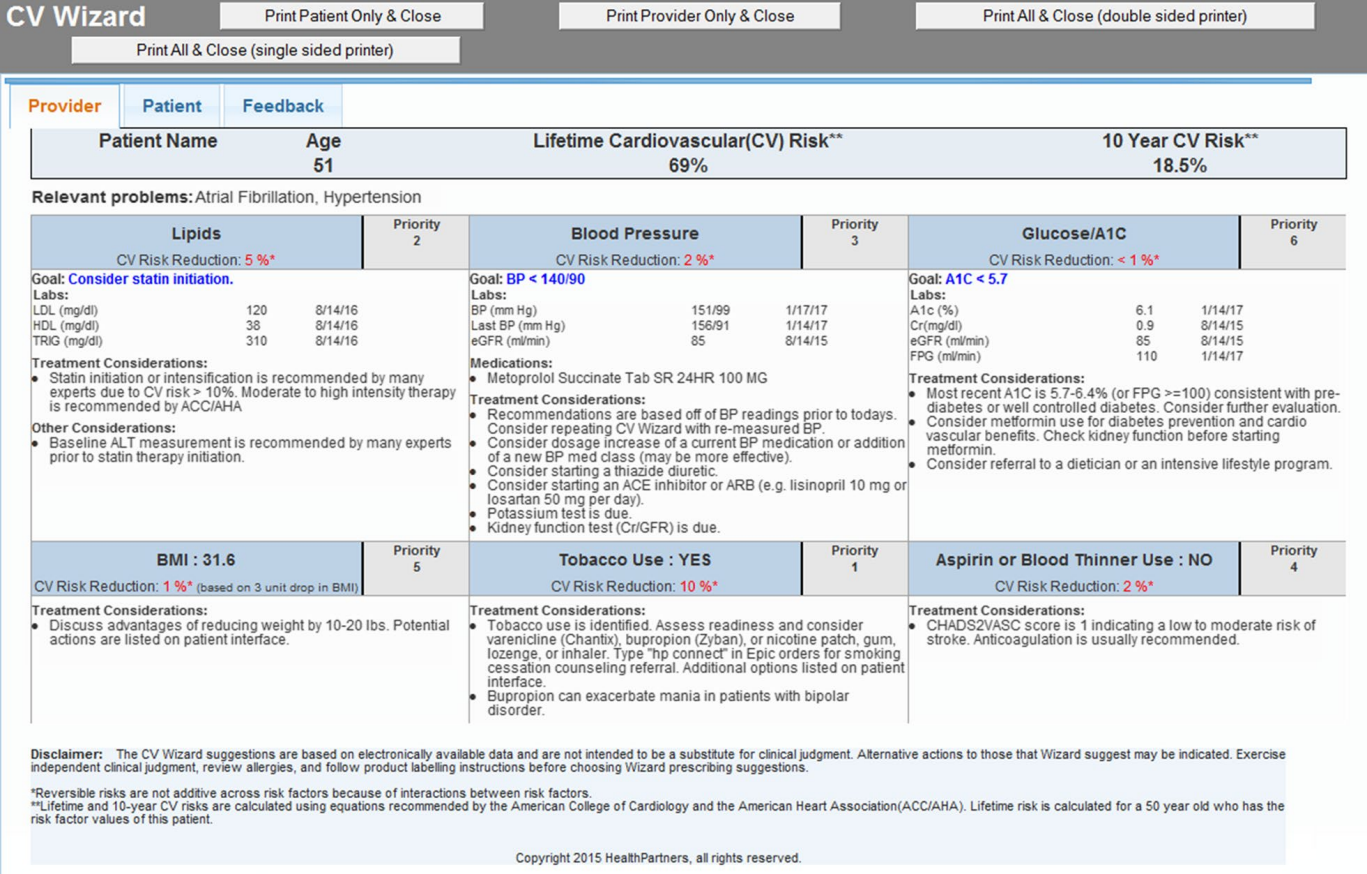
Provider view and printout of the clinical decision support system. Reprinted from Contemporary Clinical Trials, 114, Desai J, Saman D, Sperl-Hillen JM, Pratt R, Dehmer SP, Allen C, Ohnsorg K, Wuorio A, Appana D, Hitz P, Land A, Sharma R, Wilkinson L, Crain AL, Crabtree BF, Bianco J, O'Connor PJ, Implementing a prediabetes clinical decision support system in a large primary care system: Design, methods, and pre-implementation results, 106,686, Copyright (2022), with permission from Elsevier [20]

We conducted the surveys within a cluster-randomized control trial, previously described [19, 20], of a CDS intervention in 34 primary care clinics clusters within two study arms: (a) usual care (UC) and (b) CDS. The CDS arm of the study allowed rooming staff and PCCs to receive CDS alerts and treatment recommendations for adult patients with prediabetes and one of more uncontrolled cardiovascular risk factors. The CDS summary was a unique paper printout for patients (patient version) and another for PCCs (more technical version) that included six modifiable cardiovascular risk factors as well as patient-specific treatment recommendations around each of these risk factors if uncontrolled. Figures 1 and 2 show examples of both the patient and PCC version of the CDS printout, respectively [20]. In the CDS intervention arm, an algorithm-based, point-of-care, EMR-linked CDS tool identified adults with prediabetes and one or more uncontrolled cardiovascular risk factors (smoking, BMI, blood glucose, cholesterol, aspirin usage, and blood pressure). In the EMR, a best practice alert notified rooming staff of eligible patients and instructed them to print the CDS materials, giving the lay version to patients and placing the more technical PCC version on the patient’s exam room door prior to the PCC entering. PCCs in the UC arm clinics did not have access to the CDS and participants in the UC study arm would have met criteria for the CDS if they had visited an intervention clinic. The CDS was developed by the study team and tested at two pilot clinics with routine feedback collected from PCCs by the study team, with study design described in a previous publication [20]. The overall results of the RCT are forthcoming.

### Survey instrument

The surveys were administered through the Center for Evaluation and Survey Research at HealthPartners Institute using REDCap (Research Electronic Data Capture) and sent to eligible PCCs [21, 22]. Both surveys measured PCCs’ demographics, experiences in delivering care to adult patients at high risk of cardiovascular disease, views on shared decision making, their opinions of the EMR’s ability to assess and manage CV risk [6], and their overall perceptions of the CDS tool in managing CV risk (post-implementation survey only). As noted by Harry et al. [6], the surveys contained measures that were adapted from two validated instruments:  the Shared Decision-Making Questionnaire–physician version (SDM-Q-Doc) [23] and the System Usability Scale (SUS) [24]. Survey questions regarding experience and satisfaction with the CDS tool among PCCs in intervention clinics were developed internally by the study team.

### Data collection

The electronic surveys were administered pre-implementation (6/30/2016–8/25/2016) and post-implementation (6/12/2019–8/7/2019). For both surveys and as described by Harry et al. [6], an initial email requesting survey participation was sent to PCCs eligible for this study from primary care leadership at Essentia Health. This was followed by an email invitation including the survey link sent from REDCap, with up to eight email reminders sent to those who had not yet completed the survey. Pre-implementation and post-implementation surveys could only be taken once each through a unique link tied to a PCC's email address through REDCap. Completion of the survey implied PCC consent. Essentia Health’s Institutional Review Board reviewed this study in advance, approved it, and monitored its progress.

### Data analysis

Bivariate tests of association compared responses between pre- and post-implementation, as well as differences within the intervention and UC groups in the post-implementation survey. Tests were two-tailed, with an alpha of 0.05. Survey responses were collapsed into meaningful categories (e.g., scales from 0 to 10 where 0 = Never and 10 = Always were dichotomized into groups responding either 0–6 or 7–10). Differences in survey responses by treatment group within measurement time points (i.e., pre- and post-implementation) were assessed using Pearson's chi-squared tests and two-sample *t*-tests. Generalized linear mixed models assessed whether changes in survey responses from pre- and post-implementation differed by treatment group (i.e., treatment by time interaction). These models included random clinician intercepts to account for dependence in pre- and post-implementation survey data from clinicians and used normal or binomial distributions and identity or logit link functions, as appropriate for the survey response variable. This analysis was performed to account for the paired data arising when some PCCs completed both pre- and post-implementation surveys. Analyses were performed with SAS version 9.4 [25].

For the 10-year CVD risk score and each CVD risk factor (smoking, blood pressure, A1C, lipid, and weight), we measured PCC perceptions of the CDS’s usefulness using four-point scale items (extremely useful, very useful, somewhat useful, not useful). Measurement of PCC satisfaction also used four-point scale items (extremely satisfied, very satisfied, somewhat satisfied, not at all satisfied). The percent agree shown in Tables 3, 4 and 5 are expressed by combining responses for extremely useful/satisfied and very useful/satisfied.

We asked PCCs to rate their level of CDS use and how often they give the more comprehensive clinician version of the CDS patients on a percentage scale ranging from 0% to 100%. The percent agree shown in Table 5 are expressed by combining responses from 50% to 100%. PCCs selected either “yes” or “no” if they would recommend the CDS to colleagues. The percent agree shown in Table 5 is expressed by those who selected “yes”.

## Results

We emailed 294 PCCs an invitation to take part in the post-implementation survey, to which 131 responded (44.5% response rate). Table 1 shows PCC respondent demographics at pre- and post-implementation. Similar to pre-implementation, where 41% of PCCs practiced in a rural or small-town clinic and women comprised 60% of the sample [6], post-implementation survey respondents were largely women (58%) and practiced in rural or small-town clinics (40%). Physicians comprised the majority of respondents.

**Table 1:** Characteristics of study-eligible primary care clinician (PCC) survey respondents

Measure	Pre-implementation	Post-implementation
	n (%)	n (%)
Sample size	162 (100)	131 (100)
Age range (years)		
≤ 34	27 (20)	25 (23)
35–44	35 (26)	35 (32)
45–54	27 (20)	22 (20)
55–64	36 (27)	21 (19)
≥ 65	9 (7)	5 (5)
Clinic RUCA code		
Metro/micro	99 (61)	78 (60)
Small town/rural	63 (39)	53 (40)
Days a week sees patients		
3	29 (18)	27 (21)
4	73 (45)	53 (41)
5	60 (37)	51 (39)
Provider type		
Nurse practitioner	47 (29)	36 (28)
Physician assistant	23 (14)	21 (16)
Family practice physician	72 (44)	59 (45)
Internal medicine physician	20 (12)	11 (9)
Other		2 (2)
Race		
American Indian	3 (2)	Do not have
Asian	5 (3)	Do not have
Black	2 (1)	Do not have
White	148 (91)	Do not have
Unknown	4 (3)	Do not have
Sex		
Female	95 (59)	79 (60)
Male	61 (38)	47 (36)
Missing	6 (4)	5 (4)

### CDS impact on PCC management of CV risk factors in prediabetes patients

We found no significant differences between pre-and post-implementation survey responses, and no significant differences within the post-implementation survey respondents for intervention and UC groups (Table 2). Only 52% of intervention and 45% of control respondents reported always using CV risk calculations with patients. However, over 90% of post-implementation respondents reported they are well prepared to discuss dietary and physical activity for preventing diabetes or reducing CV risk. Only 38% of intervention respondents and 52% of control respondents believed it was easy to follow aspirin guidelines to determine if a patient would benefit for primary prevention (non-significant).

**Table 2:** Provider experience in delivering care to adult patients at high risk of cardiovascular disease or diabetes

Response	Measure	Question	Pre-implementation		*p*-value	Post-implementation		*p*-value	Treatment by time interaction *p*-value
			CDS	UC		CDS	UC		
			n = 102	n = 60		n = 78	n = 53		
		Please mark the response that best fits your experience in providing care to adult patients who are at high risk of cardiovascular disease or diabetes							
0–10	Always	At typical clinic visits for non-acute illnesses, how often do you discuss CV risk reduction with your patients? n (% Always)	74 (72.5)	50 (83.3)	0.1177	53 (70.7)	39 (75.0)	0.5910	0.2449
0–10	Always	At these typical clinic visits, how well prepared do you feel to prioritize CV risk factors and discuss them with your patients? n (% Always)	79 (78.2)	47 (78.3)	0.9863	60 (80.0)	42 (82.4)	0.7413	0.3152
0–10	Always	At these typical clinic visits, how often do you use CV risk calculations while seeing patients? n (% Always)	46 (45.1)	17 (28.3)	0.0345	39 (52.0)	23 (45.1)	0.4469	0.3990
		When you use a CV risk calculator for patient care, which source do you use most often?			0.3465			0.9029	
		Use a smartphone-based calculator (i.e., an App), n (%)	18 (17.6)	11 (18.3)		9 (11.8)	7 (13.5)		
		Use a link within the Epic EMR to a CV risk calculator, n (%)	61 (59.8)	30 (50.0)		52 (68.4)	33 (63.5)		
		Use a web-based calculator (not linked through the Epic EMR), n (%)	6 (5.9)	8 (13.3)		8 (10.5)	4 (7.7)		
0–10	Always	At typical clinic visits for non-acute illnesses, how often do you discuss prevention of diabetes with your patients? n (% Always)	61 (60.4)	42 (70.0)	0.2197	43 (57.3)	33 (63.5)	0.4885	0.6865
0–100%		What percentage of the time, from 0% to 100%, do you feel patients initiate conversations with you about risk factors for developing diabetes or heart disease? (95% Cl)	(23.6, 31.4)	(21.2, 31.1)	0.6707	(24.1, 33.0)	(18.9, 30.4)	0.2748	0.6539
0–10	Easy	At these typical clinical visits, how easy is it to follow aspirin guidelines to determine if a patient will benefit from taking aspirin for primary prevention (e.g., US Preventive Services Task Force recommendations)? n (% Easy)	45 (44.6)	28 (47.5)	0.7221	29 (38.2)	27 (51.9)	0.1231	0.7072
0–10	Well prepared	At typical clinic visits for patients with prediabetes, how well prepared do you feel to discuss metformin or other glucose-lowering medications for preventing diabetes or reducing CV risk? n (% Well prepared)	67 (65.7)	43 (71.7)	0.4311	64 (84.2)	40 (80.0)	0.5425	0.4500
0–10	Well prepared	At typical clinic visits for patients with prediabetes, how well prepared do you feel to discuss dietary and physical activity recommendations for preventing diabetes or reducing CV Risk? n (% Well prepared)	96 (95.0)	52 (88.1)	0.1092	74 (97.4)	45 (90.0)	0.0773	0.6382
0–10	Important	How important do you feel it is to screen adult patients at risk for prediabetes? n (% Important)	93 (91.2)	56 (93.3)	0.6256	72 (94.7)	46 (92.0)	0.5377	0.8049
0–10	Important	If a patient is in the prediabetes blood glucose range, how important is it to provide a diagnosis of prediabetes (i.e., add to the problem list and/or use ICD-10 diagnostic code)? n (% Important)	79 (78.2)	52 (86.7)	0.1831	65 (85.5)	42 (84.0)	0.8148	0.5471
0–10	Important	How important do you feel it is to talk to patients with prediabetes who are less than 65 years of age about use of metformin or other glucose-lowering medications? n (% Important)	62 (60.8)	43 (71.7)	0.1613	55 (73.3)	32 (64.0)	0.2664	0.4503

#### CDS impact on shared decision making with prediabetes patients

A significantly greater proportion of intervention respondents reported shared decision making with their patients compared to UC clinic respondents in the post-implementation survey (96% vs. 83%, respectively *p* = 0.0163) (Table 3). Most PCC respondents in both study arms believed they precisely explained advantages and disadvantages of treatment options to their patients, and most agreed they ask their patients which treatment the patient prefers, with no significant differences between pre- and post-implementation surveys. A greater proportion of intervention clinicians (88%) reported wanting to know how patients wanted to be involved in making decisions than UC clinicians (78%) (*p* = 0.0616).

**Table 3:** Provider perceptions of shared decision making with adult patients at high risk of cardiovascular disease or diabetes

Response	Measure	Question	Pre-implementation		*p*-value	Post-implementation		*p*-value	Treatment by time interaction *p*-value
			CDS	UC		CDS	UC		
			n = 102	n = 60		n = 78	n = 53		
		Thinking about your most recent visit with a patient at high CV risk and where you discussed CV risk factors…							
1–5	Agree	I made clear to my patient that a decision about reducing CV risk needs to be made, n (% Agree)	89 (88.1)	50 (83.3)	0.3927	63 (84.0)	44 (91.7)	0.2176	0.7096
1–5	Agree	I wanted to know exactly from my patient how he/she wants to be involved in making that decisions, n (% Agree)	82 (81.2)	43 (71.7)	0.1609	66 (88.0)	36 (75.0)	0.0616	0.7018
1–5	Agree	I told my patient that there are different options for reducing his/her CV risk, n (% Agree)	89 (89.0)	54 (90.0)	0.8425	68 (90.7)	43 (89.6)	0.8434	0.7018
1–5	Agree	I precisely explained the advantages and disadvantages of treatment options to my patients, n (% Agree)	81 (80.2)	46 (76.7)	0.5955	64 (85.3)	40 (83.3)	0.7646	0.5647
1–5	Agree	I helped my patient understand all the information about ways to reduce CV risk, n (% Agree)	80 (79.2)	49 (81.7)	0.7054	60 (80.0)	40 (83.3)	0.6437	0.9470
1–5	Agree	I asked my patient which treatment options he/she prefers, n (% Agree)	91 (91.0)	53 (88.3)	0.5862	69 (93.2)	42 (87.5)	0.2793	0.7580
1–5	Agree	My patient and I thoroughly weighed the different treatment options, n (% Agree)	72 (71.3)	43 (71.7)	0.9589	59 (79.7)	35 (72.9)	0.3820	0.7181
1–5	Agree	My patient and I selected treatment options together, n (% Agree)	87 (86.1)	47 (78.3)	0.1999	72 (96.0)	40 (83.3)	0.0163	0.5045
1–5	Agree	My patient and I reached an agreement on how to proceed, n (% Agree)	86 (85.1)	46 (78.0)	0.2487	69 (93.2)	42 (89.4)	0.4498	0.3011
		Shared decision-making summary score, Mean ± SD	76.6 ± 13.4	75.2 ± 16.5	0.5527	78.1 ± 12.3	81.1 ± 15.1	0.2407	0.1440

Percentages calculated by combining agree and strongly agree responses from a five-point scale (strongly disagree, disagree, neither agree nor disagree, agree, strongly agree)

### Clinician perceptions of EMR’s ability to help assess and manage CV risk

A significantly greater proportion of intervention vs control PCCs (59% vs. 41%, *p* = 0.0482) agreed that the EMR decision support is easy to use and helps manage a patient’s CV risk post-implementation (Table 4). Only about half of post-implementation PCCs agreed that most clinicians could *learn to use our EMR decision support very quickly to help manage a patient’s CV risk*. Compared to pre-implementation (CDS = 26%, UC = 27%), post-implementation CDS intervention arm respondents had significantly higher changes in the rate of agreeing that the *various functions in Essentia Health’s EMR decision support were well integrated for helping manage a patient’s CV risk* (CDS = 47%, UC = 35%, *p* = 0.0352). No other significant differences were seen.

**Table 4:** Provider perceptions of EMR’s ability to help assess and manage CV risk of patients at high risk of cardiovascular disease or diabetes

Response	Measure	Question	Pre-implementation		*p*-value	Post-implementation		*p*-value	Treatment by time interaction *p*-value
			CDS	UC		CDS	UC		
			n = 102	n = 60		n = 78	n = 53		
		…describe your reactions to your EMR’s ability to help assess and manage the CV risk of patients at high risk for diabetes or cardiovascular disease							
1–5	Agree	I would like to use our EMR decision support more often to help better manage a patient’s CV risk, n (% Agree)	79 (80.6)	43 (75.4)	0.4480	49 (68.1)	33 (68.8)	0.9361	0.1158
1–5	Agree	Our EMR decision support is unnecessarily complex for helping me manage a patient's CV risk, n (% Agree)	36 (37.1)	18 (31.6)	0.4871	21 (29.2)	14 (29.2)	1.0000	0.6957
1–5	Agree	Our EMR decision support is easy to use for helping me manage a patient’s CV risk, n (% Agree)	37 (37.8)	19 (33.3)	0.5805	42 (59.2)	20 (40.8)	0.0482	0.0646
1–5	Agree	I would need assistance to be able to use our EMR decision support to help me manage a patient's CV risk, n (% Agree)	39 (40.2)	25 (43.9)	0.6569	21 (29.6)	19 (38.8)	0.2934	0.8244
1–5	Agree	The various functions in our EMR decision support are well integrated for helping to manage a patient’s CV risk, n (% Agree)	25 (25.5)	15 (26.8)	0.8621	34 (47.2)	17 (34.7)	0.1707	0.0352
1–5	Agree	There is too much inconsistency in our EMR’s decision support ability to help manage a patient’s CV risk, n (% Agree)	14 (14.4)	13 (22.8)	0.1870	18 (25.0)	8 (16.3)	0.2542	0.3287
1–5	Agree	Most providers can learn to use our EMR decision support very quickly to help them manage a patient's CV risk, n (% Agree)	43 (44.8)	23 (40.4)	0.5918	37 (52.1)	28 (57.1)	0.5867	0.6287
1–5	Agree	Our EMR decision support is very cumbersome/awkward to use for helping manage a patient’s CV risk, n (% Agree)	33 (34.0)	19 (33.3)	0.9306	16 (22.9)	14 (29.2)	0.4394	0.4710
1–5	Agree	I feel confident using our EMR decision support to help manage a patient’s CV risk, n (% Agree)	40 (40.8)	22 (38.6)	0.7856	36 (50.0)	18 (37.5)	0.1775	0.1781
1–5	Agree	I need to learn a lot of things before I could use our EMR decision support to help manage a patient’s CV risk, n (% Agree)	33 (34.0)	16 (28.1)	0.4440	15 (20.8)	11 (22.9)	0.7861	0.7931
		System Usability Scale Summary Score, Mean ± SD	54.0 ± 16.7	52.6 ± 18.9	0.6294	58.9 ± 19.8	57.4 ± 17.7	0.6833	0.1654

Percentages calculated by combining agree and strongly agree responses from a five-point scale (strongly disagree, disagree, neither agree nor disagree, agree, strongly agree)

### PCC perceptions of the CDS Wizard to manage CV risk

Among post-implementation respondents in the intervention clinics (n = 78), 73% agreed the CDS intervention improved CV risk factor control (despite only 38% agreeing that they were satisfied with the CDS), but only 42% reported frequently using the CDS as a tool to help care for patients. Among CDS intervention clinic PCCs, 78% reported the American College of Cardiology/American Heart Association (ACC/AHA) Pooled 10-year CV disease risk score calculated within the CDS to be useful. In contrast, only 46% found the smoking domain useful, 50% found the blood pressure domain useful, 46% found the glucose domain useful, 51% found the lipid domain useful, 42% found the weight domain useful, and 42% found the aspirin domain useful. Similar results were found regarding how PCCs responded to the usefulness of the CDS Wizard in getting patients to take action on smoking (16%), blood pressure (31%), glucose (29%), lipids (33%), weight (20%), and aspirin (31%). Only 40% and 42% responded that the Wizard saves time talking about CV risk reduction and preventing diabetes, respectively, while 53% reported the Wizard actually increases the duration of appointments with patients. However, about 68% reported the Wizard adds value to patient clinic visits. Finally, 78% reported the 10-year American College of Cardiology atherosclerotic CVD risk score calculated by the Wizard to be useful (Table 5).

**Table 5:** Provider perceptions of CDS Wizard to manage CV risk of patients at high risk of cardiovascular disease or diabetes

Response	Measure		Post-implementation
			n (%)
			78 (100)
1–4	Agree	The Wizard is a tool that helps improve risk factor control of my patients at high risk of diabetes or cardiovascular disease, n (% agree) ^a^	44 (73.3)
1–4	Agree	The Wizard saves me time talking about preventing diabetes with my patients, n (% agree) ^a^	25 (41.7)
1–4	Agree	The Wizard saves me time talking about cardiovascular risk reduction with my patients, n (% agree) ^a^	24 (40.0)
1–4	Agree	The Wizard increases the duration of the appointments with my patients, n (% agree) ^a^	32 (53.3)
1–4	Agree	The Wizard adds value to my patient clinic visits, n (% agree)^a^	40 (67.8)
1–4	Well	How well do you understand which clinical conditions cause Wizard to prompt your rooming staff to print the patient and provider displays for a given visit? n (% agree)^b^	21 (35.0)
1–4	Useful	When you use the Wizard, how useful do you find the 10-year ASCVD risk score? n (% agree)^c^	43 (78.2)
0–100%	Percent of the time	When patient has elevated CV reversible risk, what percent of time do you use the Wizard information to help care for your patient? n (% used Wizard ≥ 50% of the time)^d^	25 (41.7)
0–100%	Percent of the time	When the Wizard is printed for your patient, what percent of the time do you give them the provider version to review and/or take home? n (% used Wizard ≥ 50% of the time)^d^	16 (31.4)
1–4	Useful	The Wizard smoking domain is useful in supporting/guiding your clinical decisions with patients, n (% useful)^c^	25 (46.3)
1–4	Useful	The Wizard blood pressure domain is useful in supporting/guiding your clinical decisions with patients, n (% useful)^c^	27 (50.0)
1–4	Useful	The Wizard glucose level domain is useful in supporting/guiding your clinical decisions with patients, n (% useful)^c^	24 (46.2)
1–4	Useful	The Wizard lipid level domain is useful in supporting/guiding your clinical decisions with patients, n (% useful)^c^	27 (50.9)
1–4	Useful	The Wizard weight domain is useful in supporting/guiding your clinical decisions with patients, n (% useful)^c^	22 (41.5)
1–4	Useful	The Wizard aspirin use domain is useful in supporting/guiding your clinical decisions with patients, n (% useful)^c^	22 (41.5)
1–4	Useful	The Wizard smoking domain is useful in getting patients to take action, n (% useful)^c^	8 (15.7)
1–4	Useful	The Wizard blood pressure domain is useful in getting patients to take action, n (% useful)^c^	16 (31.4)
1–4	Useful	The Wizard glucose level domain is useful in getting patients to take action, n (% useful)^c^	15 (29.4)
1–4	Useful	The Wizard lipid level domain is useful in getting patients to take action, n (% useful)^c^	17 (33.3)
1–4	Useful	The Wizard weight domain is useful in getting patients to take action, n (% useful)^c^	10 (19.6)
1–4	Useful	The Wizard aspirin domain is useful in getting patients to take action, n (% useful)^c^	16 (31.4)
1–4	Satisfied	How satisfied are you with the Wizard? n (% satisfied)^b^	23 (38.3)
Y/N		I would recommend the Wizard to my colleagues, n (% yes)^e^	38 (71.7)

^a^Percentages calculated by combining agree and strongly agree responses from four-point scale (strongly agree, agree, disagree, strongly disagree)

^b^Percentages calculated by combining very and extremely responses from four-point scale (not at all, somewhat, very, extremely)

^c^Percentages calculated by combining very useful and extremely useful responses from four-point scale (not at all useful, somewhat useful, very useful, extremely useful)

^d^Percentages calculated by combining 50–100% responses from 10%-increment 0–100% scale (0, 10, 20, 30, 40, 50, 60, 70, 80, 90, 100)

^e^Percentages calculated reflects all yes responses from yes–no response options

## Discussion

PCC attitudes towards and use of modern EMR-linked and web-based point-of-care CDS systems are not well understood. In this study, we sought to expand our understanding of factors that may affect use and effectiveness of an integrated cardiometabolic CDS system in primary care settings. We previously described transportable lessons based on our experiences for modifying CDS systems [26]. We reiterate the value of gaining “front-line key informant input early—and sustain[ing] those relationships”, as well as better estimation of “the challenges of technology” [26]. The data we report here suggests there are many opportunities to improve CDS design, implementation, and use in primary care settings.

Most PCCs exposed to the CDS reported that it helped improve CV risk factor control in patients, and most felt the CDS was well integrated in the EMR. While an overwhelming majority of post-implementation intervention arm PCCs valued easy access to a patient’s ACC/AHA 10-year CV disease risk score, only about half valued treatment suggestions related to specific clinical domains such as lipid, blood pressure, smoking, or weight management. These differences in PCC valuations of certain parts of the CDS over others suggests specific directions for future CDS development and preferred clinical use. For example, detailed information on specific treatment recommendations may not be as valuable a function in primary care CDS as directing patient and PCC attention to clinical issues, such as blood pressure or lipid management, that merit attention.

Evidence-based clinical algorithms have the potential to improve decision making, enhance shared decision making, and save time [9, 13, 18, 27, 28]; however, a majority of PCCs in our survey did not agree that the tool saved time talking about CV risk or diabetes, and about half responded that CDS use increased the duration of a clinical encounter. This finding points to the need for further refinements and focusing of CDS content, as well as flexible integration of CDS use in the clinic workflow—a workflow that may vary across PCCs even within the same clinic. Using workflow integration analysis, Salwei et al. [29] recently described 25 components of workflow integration of a CDS in the emergency department and proposed a checklist so future CDS teams can consider workflow integration. This type of analysis may prove to be relevant in improving CDS utilization in primary care as well as other departments in healthcare [29].

Although most respondents were not fully satisfied with the Wizard CDS system, over 70% would recommend it to their colleagues. Though a seemingly contradictory finding, this is evidence of a clear need for CDS improvement rather than eliminating it altogether, and much greater investigation into why PCCs were so dissatisfied. Moreover, the study team identified that while the CDS was routinely printed for patients and PCCs as we established an 80% print goal, it was often not actually utilized within the patient visit for several reasons: printing and technological issues, CDS firing too often at visits because of the addition of a cancer clinical domain due to an additional study, PCCs not having enough time, and non-optimal placement of printers outside of patient rooms [26, 30, 31]. A forthcoming manuscript reporting on our primary outcome will explore this further.

However, in prior studies of similar CDS systems, we have found similar levels of PCCs who would recommend the CDS system to their colleagues [27, 28, 32]. The consistency of this finding across several studies suggests broad PCC support for use of CDS in primary care, as well as the need for ongoing improvement of CDS tools designed for use in primary care settings. As patients are also primary users of the tool, a forthcoming study will be examining patient satisfaction with the CDS tool.

Several factors limit the interpretation of these data. The sample size was limited as was the survey response rate at 44.5% at post-implementation. Most of the randomized clinics were located in rural areas or small towns, and generalizability of results to large urban settings should be done with caution. PCC perceptions were related to only one CDS system, and all PCCs in both CDS intervention and UC clinics had some access to simple EMR-based prompts and reminders. Also, many of our survey questions around satisfaction were developed internally and not from a standardized survey instrument. Despite these limitations, our results suggest widespread PCC interest in and use of CDS systems in primary care and suggest specific enhancements that may be considered to improve CDS design and clinical content in primary care settings.

## Conclusions

PCCs in UC clinics reported confidence in their ability to manage major CV risk factors in high-risk patients such as those with prediabetes. PCCs in CDS intervention clinics reported that easy access to 10-year CVD risk estimates was useful and believed that CDS improved CV risk factor management. However, they did not place high value on domain-specific care suggestions and reported that CDS use takes too much time. Despite their perceptions of limited CDS usefulness and lack of satisfaction, about 72% would recommend use of this CDS system to their colleagues, suggesting that improving the design and content of CDS systems to support chronic disease care in primary care settings would be valued by PCCs. Future iterations of CDS systems designed for use in primary care should take into account these findings to guide improvements.

## Data Availability

The datasets generated and/or analyzed during the current study are not publicly available due to privacy but are available in deidentified form from the corresponding author on reasonable request.

## Abbreviations

CVD: Cardiovascular disease
CDS: Clinical decision support
PCC: Primary care clinician
CV: Cardiovascular
UC: Usual care
BMI: Body mass index
EMR: Electronic medical record
REDCap: Research electronic data capture
SDM-Q-Doc: Shared decision-making questionnaire–physician version
SUS: System Usability Scale
